# The relationship between catastrophic health expenditure and health-related quality of life

**DOI:** 10.1186/s12939-018-0883-0

**Published:** 2018-11-14

**Authors:** Seung Hyun Kang, Yeong Jun Ju, Hyo Jung Yoon, Sang Ah Lee, Woorim Kim, Eun-Cheol Park

**Affiliations:** 1 Department of Public Health, Graduate School, Yonsei University, Seoul, Republic of Korea; 2 Institute of Health Services Research, Yonsei University, Seoul, Republic of Korea; 3 Department of Preventive Medicine and Institute of Health Services Research, Yonsei University College of Medicine, 50 Yonsei-ro, Seodaemun-gu, Seoul, 03722 Korea

**Keywords:** Catastrophic health expenditure, Health-related quality of life, Out-of-pocket health payment, Chronic disease

## Abstract

**Objectives:**

The objective of our study was to investigate the relationship between catastrophic health expenditure (CHE) and health-related quality of life (HRQoL) in general population.

**Methods:**

We used Korean Health Panel Survey data from 2011 to 2013, which included data from 8850 baseline participants of 19 years of age or older. We defined CHE as total annual out-of-pocket health payment that was 40% greater than the household’s capacity to pay. HRQoL was measured using the EuroQol-visual analogue scale (EQ-VAS). We used generalized estimating equations to perform a longitudinal regression analysis.

**Results:**

A total of 4.5% of the participants (*n* = 398) experienced CHE. Those with CHE tended to have a lower EQ-VAS index score compared with those without CHE (β: − 1.34, *p* = 0.013). A subgroup analysis revealed that individuals experiencing CHE had significant decreases as the number of chronic diseases increased (three or more, β: − 1.85, *p* = 0.014).

**Conclusions:**

Catastrophic health expenditure influences HRQoL, which was more pronounced in patient with chronic disease. The efforts should focus on people who suffer from excessive health expenditures and chronic diseases.

## Introduction

Health-related quality of life (HRQoL) is a multi-dimensional concept that includes domains related to physical, mental, emotional, and social functioning and the social context in which people live [1]. HRQoL is an important outcome used in a variety of medical research disciplines to ascertain aspects of well-being in settings of health and disease. Since 1949, the World Health Organization (WHO) has emphasized the importance of HRQoL [2]. The WHO’s ‘Healthy People 2020’ initiative emphasizes HRQoL as one of its four overarching goals [3].

As factors affecting HRQoL vary, HRQoL has been analyzed as an outcome in a variety of populations and settings [4]. Previous studies to clarify the factors affecting HRQoL have generally considered physical functioning (e.g., overall physical health, physical functioning, pain, fatigue), disease-specific (e.g., cancer, chronic disease), health care service use (e.g., unmet healthcare needs) factors as relevant [5–9]. In particular, not only burden for clinical status but also socio-economic burden can also affect HRQoL. For example, financial hardship was associated with degenerated physical and psychological heath, thereby exacerbating HRQoL [10]. In addition, financial hardship that occurs after receiving hematopoietic cell transplantation was associated with worse quality of life and exacerbated perceived stress [11]. Studies on this topic have focused primarily on specific disease with high medical expenditures such as cancer. On the other hands, in line with the increasing trend of socio-economic burden of chronic disease such as non-communicable disease [12], study reported that high medical expenditure including out-of-pocket expenditure in type 2 diabetic patients was associated to poor health-related quality of life [13]. Especially, household members with chronic illness are the major factors affecting financial catastrophe, financial hardship of healthcare was greater for subjects affected by chronic disease than those unaffected [14–16]. Hence, catastrophic healthcare expenditure which implies a financial hardship due to medical expenditure may impact on health-related quality of life. Only a few studies have addressed this issue. This topic is especially relevant to countries with concerns about health-related life satisfaction and health service utilization.

In Korea, subjective health satisfaction is the lowest among all OECD countries. According to an OECD report, only 35.1% of Koreans ≥15 years of age believe their health condition to be “good”. This value is approximately one-half the OECD average of 69.2%. Hence, it is necessary to examine health-related life satisfaction issues in terms of promotion and identification of the factors that affect HRQoL. In addition, a national health insurance system provides universal healthcare coverage in Korea, but there are barriers to medical care access because high out-of-pocket payments (OOP) cause catastrophic health expenditures (CHEs). Overall, the South Korean OOP payment for healthcare is the highest among OECD countries (Korea: 4.7%; OECD average: 2.8%) [17]. Korea also has a relatively greater proportion of households with catastrophic expenditures [17, 18].

Therefore, the present study used longitudinal data and analysed the effects of catastrophic health expenditure on HRQoL in the general population. In addition, we examined the relationship between CHE and HRQoL by number of chronic disease.

## Materials and methods

### Study population

We used raw data from the Korean Health Panel Study (KHPS) conducted in 2008 and 2013. The group of study participants was a nationally representative sample of Korean. The KHPS is a panel survey conducted annually by the Korean Institute for Health and social Affairs in conjunction with the National Health Insurance service on a nationally representative sample of South Korean household. Households are selected using a stratified multistage probability sampling design in order to select nationwide subjects. The KHPS comprised three parts—household, individual, and case-based sections—all of which were performed by trained medical staff through a computer assisted personal interviewing. The household survey included questions about general characteristics, living expenses, pharmaceutical product purchases, and private health insurance with associated premiums. The individual survey considered the demographic characteristics of the subjects. The case-based survey was designed only for individuals with chronic diseases and those receiving inpatient treatment, outpatient treatment, or emergency-service utilization. We used data from all three surveys. Detail of the datasets are available at https://www.khp.re.kr:444/eng/main.do.

**Fig. 1:**
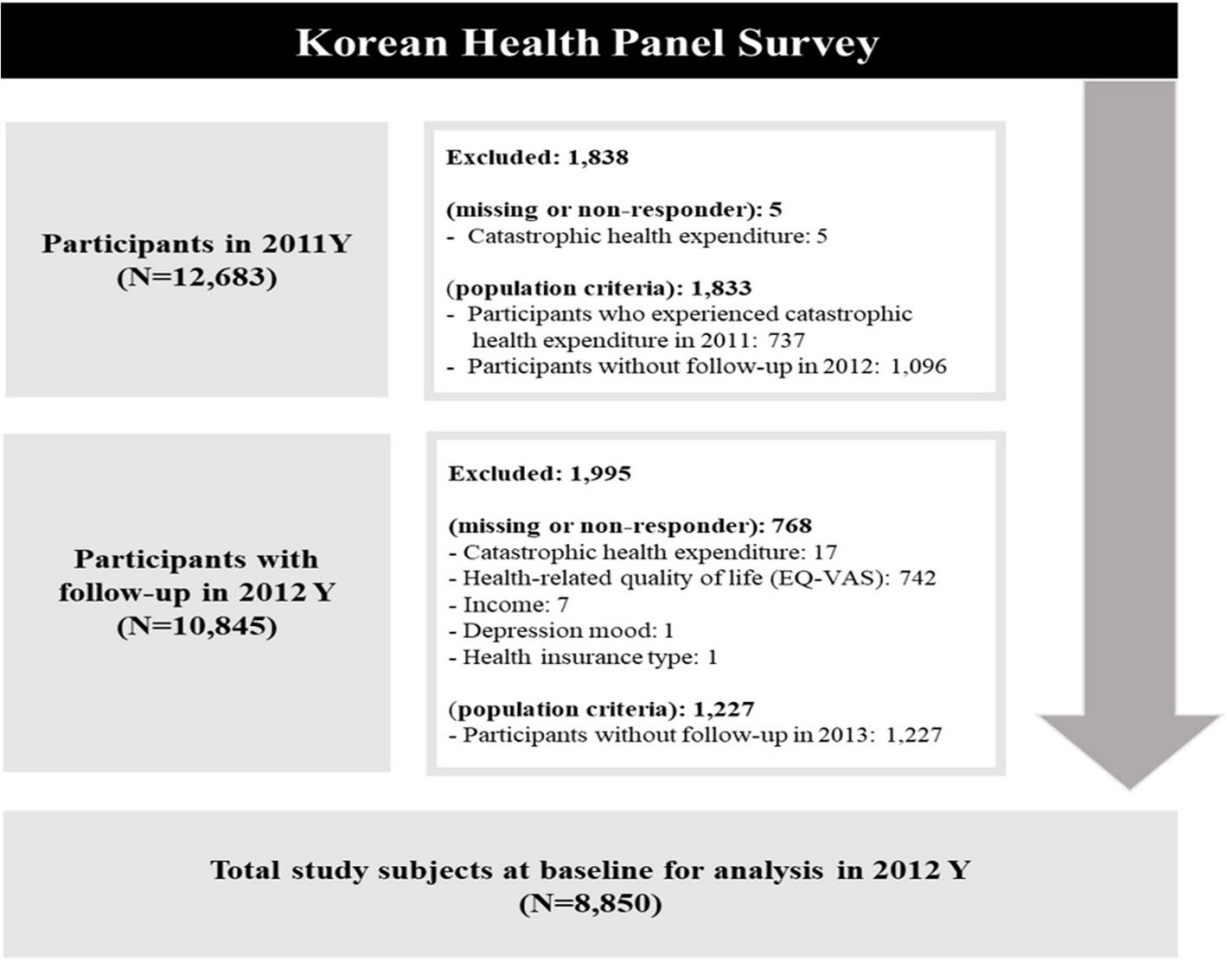
Flow chart of participant selection process

The KHPS began in 2008, but the monthly food expense variable used to calculate CHE was recorded beginning in 2011. Thus, we used data from the KHPS between 2011 and 2013. In addition, KHPS was released to raw data until 2015, but EQ-VAS was not measured in 2014 and 2015, so it was not used in the analysis. Our sample was restricted to individuals aged 19 years or older. To analysis only newly onset catastrophic health expenditure, we excluded respondents who responded that they experienced catastrophic health expenditure in 2011. Of the 12,683 in 2011, subjects with catastrophic health expenditure and without follow-up in 2012 were not included in the analysis; 1838 participants were excluded. Then 10,845 subjects were followed up in 2012. Of the 10,845 subjects in 2012, after excluding subjects with any missing values or without follow-up in 2013, a total of 8850 subjects remained in this study. Thus, baseline included 8850 subjects with a 2-year follow-up (see details in Fig. 1).

We used public KHPS data, which did not include any information that could be used to identify individuals. The survey’s design and methods conformed to local regulations and Declaration of Helsinki standards. The data, and the permission to use and analyze the data, were provided by KIHASA.

### Measures

#### Health-related quality of life

We measured HRQoL using the EuroQoL-visual analogue scale (EQ-VAS) index. EQ-VAS is a self-rated health questionnaire presented as a vertical visual “thermometer” with end-point values of 100 (best imaginable HRQoL) and 0 (worst imaginable HRQoL). Higher scores correspond to a higher HRQoL.

#### Catastrophic health expenditure

We used the WHO standard threshold to define CHE as a total annual OOP health payment that is 40% greater than the household’s capacity to pay [19].

The capacity to pay is defined as the economic power to which a household can purchase a particular goods or service, except for expenditure necessary for a living. We used Xu et al.’s measure to define the capacity to pay as the amount of money available after excluding food expenses [20]. The monthly food expenditure was deducted from the monthly living expenditure, and then multiplied by 12 to obtain the yearly payment conversion.

The OOP was defined as medical expenses borne by the family at the time of receiving the health care services as defined by the WHO [19]. The annual OOP health payment was calculated by including medical and drug costs resulting from emergency, outpatient care, and hospitalization, services. Indirect medical costs (e.g., including transportation or nursing costs) were excluded from the calculation.

$$ \mathrm{CHE}=\frac{\mathrm{household}\ \mathrm{out}-\mathrm{of}-\mathrm{pocket}\ \mathrm{health}\ \mathrm{expenditure}}{\mathrm{household}\ \mathrm{expenditure}\ \left(\mathrm{excluding}\ \mathrm{food}\ \mathrm{expenses}\right)}>40\% $$

#### Covariates

We included several demographic, socioeconomic, and health-related variables as covariates. The demographic variables included sex and age. The socioeconomic variables included education level (elementary school or below, middle school or high school, college or above), economic activity (employed, unemployed), family constitution (living alone, couple, couple with child, more), health insurance type (health insurance, medical aid), and income (low, low-middle, middle, middle-high, high). The health-related variables were the number of chronic diseases, disability (yes, no), how perceive health status (good, bad), depressive mood during the past 2 weeks (present, absent). Survey year was included as a covariate.

#### Statistical analysis

Descriptive statistics were presented as number of subjects and proportions. Univariate analyses were performed to compare the mean EQ-VAS score values for two groups using *T*-test as well as analysis of variance (ANOVA) were performed to compare the mean EQ-VAS score values among the three or more groups. We evaluated the relationship between catastrophic health expenditure and HRQoL using a generalized estimating equation (GEE) model that was an extension of the quasi-likelihood approach used to analyses longitudinal correlated data [21]. The GEE model was used for analyzing longitudinal data, as it accounted for time variation and correlations between repeated measurements. In details, the statistical analyses were performed using the GENMOD procedure. It computes robust standard error estimates by default and accounts for the correlations among repeated measurements [22]. All independent variables were adjusted. Finally, a subgroup analysis was performed to evaluate a possible association between catastrophic health expenditure and HRQoL stratified by number of chronic disease.

The analyses were performed using SAS 9.4 (SAS Institute, Cary, North Carolina, USA) and *p*-values were two-sided and considered significant at *p* < .05.

## Results

In our study, 8850 subjects were included to assess the association between catastrophic health expenditure and health-related quality of life. Table 1 shows the baseline characteristics of the study population. Among the 8850 subjects, 4.5% (*n* = 398) experienced catastrophic health expenditure. The mean baseline EQ-VAS score was 70.76 ± 15.39. The EQ-VAS score value was lower for those who experienced CHE (64.46 ± 17.56) compared with those who did not experience CHE (71.06 ± 15.21). Lower scores indicated more severe status in HRQoL.

**Table 1:** General characteristics of study population at baseline in 2012

Variables	EQ-VAS						
	N	(%)	Mean ± S.D			*t / F value*	*p*-value
Catastrophic expenditure						−7.37	<.0001
Yes	398	(4.5)	64.46	±	17.56		
No	8452	(95.5)	71.06	±	15.21		
Sex						8.42	<.0001
Male	3834	(43.3)	72.32	±	14.72		
Female	5016	(56.7)	69.57	±	15.78		
Age						104.34	<.0001
19~ 29	733	(8.3)	75.19	±	14.60		
30~ 39	1421	(16.1)	73.56	±	14.55		
40~ 49	2057	(23.2)	73.65	±	13.37		
50~ 59	1693	(19.1)	70.86	±	14.55		
60~ 69	1503	(17.0)	68.36	±	16.03		
70+	1443	(16.3)	64.03	±	16.88		
Education level						281.25	<.0001
Elementary or below	1912	(21.6)	64.16	±	16.87		
Middle/high school	3955	(44.7)	71.14	±	14.85		
College or above	2983	(33.7)	74.49	±	13.63		
Economic status						10.29	<.0001
Employed	5498	(62.1)	72.11	±	14.46		
Unemployed	3352	(37.9)	68.55	±	16.56		
Income						117.73	<.0001
Low	1243	(14.0)	63.41	±	17.67		
Middle-low	1687	(19.1)	69.08	±	15.39		
Middle	1866	(21.1)	71.25	±	14.82		
Middle-high	1987	(22.4)	72.56	±	14.56		
High	2067	(23.4)	74.38	±	13.41		
Health insurance type						12.17	<.0001
Health insurance	8448	(95.5)	71.30	±	14.96		
Medical aid	402	(4.5)	59.39	±	19.34		
Family constitution						79.65	<.0001
Living alone	667	(7.5)	66.46	±	17.12		
Couple	1792	(20.3)	68.06	±	16.37		
Couple with children	4803	(54.3)	73.00	±	14.11		
More	1588	(17.9)	68.87	±	16.06		
Number of chronic disease						225.56	<.0001
0	3180	(35.9)	74.92	±	13.73		
1	1794	(20.3)	72.41	±	14.19		
2	1252	(14.1)	70.00	±	15.11		
3+	2624	(29.7)	64.96	±	16.34		
Disability						12.77	<.0001
Absent	8266	(93.4)	71.38	±	15.04		
Present	584	(6.6)	61.95	±	17.40		
Perceive health status						40.78	<.0001
Good	7478	(84.5)	73.72	±	13.20		
Bad	1372	(15.5)	54.62	±	16.41		
Depression mood						−18.53	<.0001
Present	632	(7.4)	58.11	±	18.03		
Absent	8218	(92.9)	71.74	±	14.72		
Year							
2012	8850	100.0	70.76	±	15.39		

Table 2 shows the association between CHE experiencing and HRQoL while adjusting for all independent variables. Those with CHE experiencing tended to have lower EQ-VAS index values compared with those without CHE (β: − 1.34, *p* = 0.013). A more detailed examination of the relationship between experiencing CHE and HRQoL revealed that respondents ≥70 years of age tended to have lower EQ-VAS index values compared with respondents 19~29 years of age (β: − 1.72, *p* = 0.010). An examination based on income revealed that EQ-VAS values increased as income increased (i.e., low < middle-low < middle < middle-high < high; low: − 3.26, middle-low: − 1.69, middle: − 1.75, middle-high: − 0.94). Respondents with ≥3 chronic diseases had lower EQ-VAS scores compared with those without any chronic diseases (β: − 3.11, *p* < 0.001).

**Table 2:** Results of the GEE analyzing for the effect of catastrophic health expenditure on EQ-VAS

Variables	EQ-VAS		
	β	S.E	*p*-value
Catastrophic health expenditure			
Yes	−1.34	0.54	0.013
No	Ref.		
Sex			
Male	Ref.		
Female	−0.99	0.25	0.000
Age			
19~ 29	Ref.		
30~ 39	− 0.46	0.53	0.384
40~ 49	0.44	0.51	0.387
50~ 59	−0.26	0.56	0.642
60~ 69	−0.45	0.61	0.459
70+	−1.72	0.67	0.010
Education level			
Elementary or below	−2.40	0.45	<.001
Middle/high school	−0.75	0.29	0.010
College or above	Ref.		
Economic status			
Employed	Ref.		
Unemployed	0.44	0.26	0.099
Income			
Low	−3.26	0.48	<.001
Middle-low	−1.69	0.36	<.001
Middle	−1.75	0.34	<.001
Middle-high	−0.94	0.31	0.002
High	Ref.		
Health insurance type			
Health insurance	Ref.		
Medical aid	−2.03	0.67	0.002
Family constitution			
Living alone	0.91	0.51	0.073
Couple	Ref.		
Couple with children	−1.19	0.36	0.001
More	−1.80	0.40	<.001
Number of chronic disease			
0	Ref.		
1	−1.16	0.32	<.001
2	−1.82	0.38	<.001
3+	−3.11	0.36	<.001
Disability			
Absent	Ref.		
Present	−1.96	0.52	<.001
Perceive health status			
Good	Ref.		
Bad	−14.90	0.37	<.001
Depression mood			
Present	−7.07	0.46	<.001
Absent	Ref.		
Year			
2012	Ref.		
2013	−0.83	0.18	<.001

The subgroup analysis results are shown in Table 3. Subjects with CHE and greater chronic disease (3 or more) exhibited a drastic decrease in HRQoL.

**Table 3:** Results of the GEE analyzing for the effect of catastrophic health expenditure on EQ-VAS by number of chronic disease

Variables		EQ-VAS		
		β	S.E	*p*-value
Number of chronic disease	Catastrophic expenditure			
0	No	Ref.		
	Yes	0.53	1.35	0.696
Number of chronic disease	Catastrophic expenditure			
1	No	Ref.		
	Yes	−0.54	1.50	0.719
Number of chronic disease	Catastrophic expenditure			
2	No	Ref.		
	Yes	−2.17	1.23	0.077
Number of chronic disease	Catastrophic expenditure			
3+	No	Ref.		
	Yes	−1.85	0.75	0.014

## Discussion

We found that after adjustment for multiple variables, CHE was significantly associated with degenerated HRQoL in the general population. The results of our subgroup analysis indicated that the association between CHE and HRQoL was stronger in individuals with chronic disease.

These findings can be explained by the associations between financial burden and life satisfaction. Previous studies have examined the associations between economic hardship and life satisfaction and have found that financial burden has adverse consequences on life satisfaction characteristics [23–25]. Studies of catastrophic expenditure revealed that there is a robust association between excessive expenditure for healthcare and financial strain (e.g., onset of poverty). Hence, experiencing CHE may increase financial strain and result in a deteriorating HRQoL.

Consistent with previous studies on financial hardship, cancer survivors in the USA who have financial burdens (e.g., borrowed money) are more likely to have low Physical Component and Mental Component scores and are therefore more likely to experience a depressed mood [26]. Patients in the UK who have head and neck cancer that has resulted in serious effects on household finances have poor HRQoL [27].

Populations who suffer from a chronic disease are more likely to experience CHE because medical expenditures are likely to continue for a long period. As expenditures for chronic disease treatment accumulate, individuals or households are more likely to compromise healthy lifestyle choices. For example, they cannot afford fresh fruits and vegetables, which are more expensive than processed foods [28]. Therefore, chronic disease has the potential to negatively affect health-related life satisfaction characteristics [29]. This phenomenon has been found in developing [30] countries and in the wealthiest countries in Europe [31].

Our study revealed that 4.5% of households in Korea experienced CHE. This estimate is similar to the 3.0% that the OECD reported for 2012 using Korea national statistics. This value is also the highest among OECD countries [32]. Among developed countries, only Portugal, Greece, Switzerland, and the United States have 0.5% or more of households with catastrophic-level health spending. OOP payments for healthcare can cause households to incur catastrophic expenditures [33, 34]. Therefore, this result for Korea is expected because OOP spending as a share of total health expenditure is relatively high (Korea = 36%; OECD average = 19%) [35]. High OOP payments may create barriers to medical utilization that cause delays in care, low screening rates among vulnerable people, and exacerbate inequities in health status and in health-related life satisfaction characteristics.

Even when we excluded households that experienced CHE in the most recent year, the mean EQ-VAS score at baseline was 70.8; this value was less than that of the general population of China (80.1) [36] and of the mean overall score of six European countries (77.1) [37]. South Korea currently has serious life satisfaction issues. Koreans are substantially less satisfied with their lives compared with residents of OECD countries. The ‘Better Life Index’ report presents results for eleven parameters (e.g., income, jobs, health and work-life balance); Korea ranked 29th among OECD countries in 2014 (Korea’s score: 5.8/10; OECD average: 6.6/10). The results for the self-reported health measure of health-related life satisfaction indicated that individual South Korean citizens have the least confidence in their own health condition level. Taken together, these findings indicate that effective strategies to manage HRQoL among households with CHE should be designed and implemented.

Our findings suggested that programs (e.g., medical expense assistance) that support populations who experience CHE are needed to improving the quality of life. The Korean government recently implemented the pilot catastrophic healthcare expenditure aid program. This public assistance program targets poor individuals who experience catastrophic healthcare expenditure due to major severe diseases (e.g., cancer, cardiovascular disease, rare diseases).

We suggest that countries with low financial assistance levels for healthcare should aim to reduce the barriers within the healthcare system and allocate resources to strengthen healthcare coverage and increase healthcare equity. These efforts should emphasize guarantee of healthcare services for people who suffer from excessive health expenditures and chronic disease.

This study had some limitations. First, the EQ-VAS measures current health status and CHE was measured using yearly health expenditure data. Therefore, the effects from external events might have moderated or reinforced the HRQoL results. Second, we used the EQ-VAS to measure HRQoL, which depends on the participant’s subjective perception. However, the EQ-VAS is widely used for HRQoL studies. Third, due to limitations of our data, we measured short-term effects (i.e., 2 years). Further studies of longer-term effects of CHE are needed.

Despite the limitations, this study is the first to investigate associations between CHE and HRQoL among the general Korean population. Given the high values for incidence of catastrophic healthcare expenditure and the low health-related satisfaction levels in Korea, our findings are important for health policy makers to identify solutions aimed at control of HRQoL characteristics.

## Conclusion

In conclusion, the present study found that catastrophic health expenditure was to have an effect on HRQoL. The HRQoL of participants with chronic disease was significantly worse than that of other groups. The efforts should focus on people who suffer from excessive health expenditures and chronic diseases.

## Abbreviations

CHE: Catastrophic health expenditure
EQ-VAS: EuroQoL-Visual analogue scale
HRQoL: Health related quality of life
KHPS: Korea health panel study
OOP: Out-of-pocket payments
WHO: World Health Organization
